# Distal Radial Artery Access in comparison to Forearm Radial Artery Access for Cardiac Catheterization: A Randomized Controlled Trial (DARFORA Trial)

**DOI:** 10.1155/2022/7698583

**Published:** 2022-07-15

**Authors:** Yunis Daralammouri, Zaher Nazzal, Yahya S. Mosleh, Heba K. Abdulhaq, Zafer Y. Khayyat, Yousef El Hamshary, Murad Azamtta, Ahmed Ghanim, Fateh Awwad, Sajed Majadla, Mosab Maree, Jihad Hamaida, Yahia Ismail

**Affiliations:** ^1^Department of Medicine, Faculty of Medicine and Health Sciences, An-Najah National University, Nablus, State of Palestine; ^2^Department of Cardiology, An-Najah National University Hospital, Nablus, State of Palestine; ^3^Department of Family and Community Medicine, Faculty of Medicine and Health Sciences, An-Najah National University, Nablus, State of Palestine; ^4^Department of Internal Medicine, An-Najah National University Hospital, Nablus, State of Palestine; ^5^Department of Radiology, An-Najah National University Hospital, Nablus, State of Palestine

## Abstract

**Background:**

In our clinical practice, conventional radial access has been employed routinely for coronary procedures. The distal radial artery (DRA) access site has recently emerged as a novel technique in cardiac procedures.

**Objectives:**

This study compares distal radial access to standard forearm radial access (FRA) in terms of feasibility, outcomes, and complications.

**Method:**

This prospective, randomized trial was conducted at a single center. The patients were chosen from An-Najah National University Hospital's catheterization laboratory between December 2019 and November 2020. A total of 209 patients were randomized into two groups: DRA group (*n* = 104) and FRA group (*n* = 105).

**Results:**

Access was successful in 98% of patients in both the groups. The DRA group had a longer puncture duration and a higher number of attempts (duration: 56.6 ± 61.1 s DRA *vs.* 20.0 ± 18.4 s FRA, *p* < 0.001, attempts: 1.9 ± 1.3 DRA vs. 1.2 ± 0.60 FRA, *p* < 0.001). Puncture-associated pain was greater in the DRA group (4 ± 2.2 DRA *vs.* 3 ± 2.1 FRA, *p*=0.001). There were two radial artery occlusions in the FRA group and none in the DRA group (*p*=0.139). Percutaneous coronary intervention (PCI) was performed in 26% of the DRA group and 37.1% of the FRA group. The DRA group had significantly shorter procedure times (*p*=0.006), fluoroscopy times (*p*=0.002), and hemostasis times (*p*=0.002). Over time, the learning curve demonstrated improved puncture duration and a decrease in the number of puncture attempts.

**Conclusions:**

DRA is a safe and practical alternative to FRA for coronary angiography and intervention. The overtime learning curve is expected to improve puncture-related outcomes.

## 1. Introduction

Ischemic heart disease (IHD) is the leading cause of death globally [1]. Advancements in healthcare systems, novel medications, and developed interventions such as cardiac catheterization have contributed to decreased morbidity and mortality by improving early diagnosis and treatment [2].

Access methods for cardiac angiography and interventions are debatable aspects, which have changed many times over the years. Femoral artery access was traditionally the utilized method. Later on, catheterization through the forearm radial artery access (FRA) escalated until the European Society of Cardiology (ESC) recommended the FRA approach as the preferred access in 2015 [3]. This change occurred with the publication of several studies demonstrating that the FRA for coronary angiography (CAG) and percutaneous intervention (PCI) was associated with lower mortality and vascular complications [4, 5].

In recent years, distal radial artery access (DRA) has grown popular. Its technique was introduced by Kaledin A. in 2014 and described by Kiemeneij F. for coronary angiography and interventions in 2017 [6–8]. This new technique has several advantages over FRA, including a lower risk of local complications, notably radial artery occlusion, and enhanced patient and operator comfort [9–13].

The distal radial artery runs through the radial fossa, known as the anatomical snuffbox (AS), and anastomoses to complete the deep palmer arch with the ulnar artery. Proximal to this site, the radial artery has already given its branch to the superficial palmar arch. This rich network of anastomosis is supposed to maintain blood flow to the digits [14].

DRA through the AS is a novel approach, but the future availability of this access point is still unclear. Moreover, the effectiveness of this strategy depends on the anthropometric characteristics of the population and the expertise of the operators, and it must be evaluated in real life and under varied settings. As a result, the goal of this study is to compare the safety, feasibility, and efficacy of this new technique (DRA) with FRA.

## 2. Methods

### 2.1. Study Design, Settings, and Population

This is a single-center, parallel-group, partially blinded, randomized controlled trial with a 1 : 1 allocation ratio. This study was conducted between December 2019 and December 2020 at the cardiology department of An-Najah National University Hospital (NNUH), Nablus, Palestine. Three interventional cardiologists were appointed as operators, each with experience of more than 200 PCI per year.

All patients who were hospitalized for cardiac catheterization and had palpable proximal and distal radial pulses were included in the study. Excluded subjects were those who had no palpable pulse on both radial access sites, were hemodynamically unstable, and who presented with ST-segment elevation myocardial infarction (STEMI). Patients with radial arteriovenous fistula (AVF) for hemodialysis and had previous coronary artery bypass grafting (CABG) using the left or right radial artery, left internal mammary artery (LIMA), right internal mammary artery (RIMA) or both were also eliminated since the access side was confined to the right or left upper limb and thus could not be assigned to randomization. Patients with the Raynaud phenomenon, previously occluded radial artery, incomplete palmar arch, and lymphedema were also excluded.

### 2.2. Sample Size, Randomization Type, and Sequence Generation

The initial success rate of the FRA approach was predicted to be between 90 and 97%; therefore, after considering the clinically acceptable range, a noninferiority margin of 7% was selected. Using the PASS 15.0 software and this noninferiority margin with a significance level of 0.05 and power of 90%, we calculated a minimum sample size of 102 participants for each arm. To account for a 10% dropout rate, 224 participants were needed.

A total of 212 patients were assigned to left DRA or FRA at random (ratio 1 : 1). To maintain balance, randomization was performed with a set block size of 4, according to a computer-generated randomization list developed with Excel Software.

### 2.3. Allocation Concealment and Implementation

The operating cardiologist evaluated the patients who were scheduled for catheterization for eligibility. In the catheterization lab, included and consented participants were randomly assigned to one of the two groups: left DRA or FRA. The operator would draw a card from a one-way, nonopaque plastic container with the access site printed on it from the inside in a sequentially ordered sequence. According to the randomization list, a nonclinical investigator had already prepared and filled the plastic container.

### 2.4. Study Procedure and Tools

All patients were covered with sterile drapes after disinfection with povidone-iodine. The left hand is placed on the right side of the groin with the dorsal surface of the hand upward. The right hand was fixed on a board secured underneath the shoulder with a wrapped towel under the wrist. The operator was positioned on the patient's right side, preparing for a left DRA or right FRA puncture.

The radial artery was initially brought to the surface for the DRA puncture by having the patient grasp his thumb towards the palm. After a local anesthetic injection of 1–3 ml of 2% lidocaine HCL subcutaneously, the needle was pointed towards the site of the greatest pulse. Following artery puncture, a 0.018-in. soft, flexible metallic wire was gently introduced while retaining the patient's wrist's semi-abducted and extended position.

**Figure 1:**
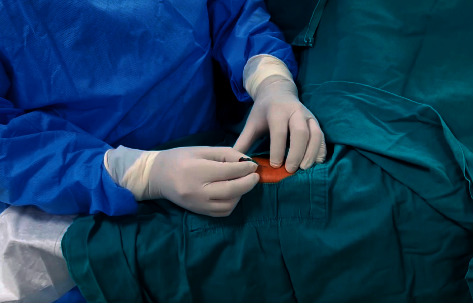
Palpation and puncture of the distal radial artery.

Then a 6-French radial hydrophilic sheath was inserted into the DRA (Figure 1). For the FAR puncture, the radial artery was punctured at a 30 to 45° angle, 1 cm proximal to the radial styloid process. To prevent radial spasm and thrombosis, all patients received a combination of weight-adjusted unfractionated heparin (40 to 70 U/kg up to 5000) and 200 ug of nitroglycerine.

**Figure 2:**
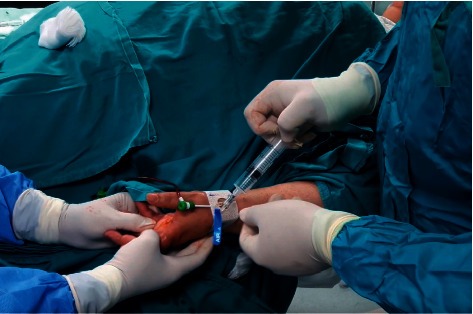
Hemostatic device is inflated before complete removal of the sheath.

At the end of the procedure, a compression band—TR Band in the FRA group and Safeguard band in the DRA group—is placed and inflated with air, and the sheath is entirely withdrawn to ensure no bleeding occurs (Figure 2). A pulse oximeter was placed on the index finger while inflating the band to ensure adequate hand blood flow. From band insertion to removal, the duration of hemostasis is documented. The compression band was deflated every 15–30 minutes to achieve proper hemostasis and avoid radial occlusion. The researchers evaluated patients for postprocedural pain and access site problems such as hematoma and bleeding. Within 24 hours, the patients were evaluated with a Doppler ultrasound of the radial artery to check for obstruction of the radial artery.

### 2.5. Endpoints

The study's primary endpoint is the success of the access puncture, which is defined as sheath insertion in the radial artery. Failed access is defined as exceeding a total of six puncture attempts or being unable to proceed wire; in these circumstances, a crossover to another access is required. Other study endpoints were puncture time, which is the time in seconds between the first and successful puncture attempt.

The procedure time was calculated in minutes, beginning with the insertion of the sheath and ending with its removal. Fluoroscopy time and radiation dose were measured by the radiological device in minutes and by milligray (mGy), respectively. The duration in minutes between the application of the compression band and its removal when there is no blood flowing following deflation is referred to as the hemostasis “compression” time.

Puncture pain and postprocedural pain were assessed by the numerical rating scale (NRS) for pain. It is an 11-point subjective scale (0–10), where 0 refers to no pain, 1–3 for mild pain, 4–6 for moderate pain, and 7–10 for severe pain [15].

Arterial spasm was assessed by the operator in terms of the difficulty in inserting the wire during the procedure. The hematoma was defined using an easy-to-use hematoma scale. Ischemic changes to the hand are noted postoperatively by examination for clinical features such as pallor, absence of pulse, pain, cold, paresthesia, or paralysis.

Complications such as pseudoaneurysm, AVF formation, and radial artery dissection were assessed by Doppler US. In addition, radial artery eversion or perforation was recorded by inspection. All were evaluated within 24 hours after the procedure. Doppler ultrasound was used to detect radial artery (RAO) within 24 hours, and it was repeated after 2 weeks for individuals who had an occluded radial artery during the first 24 hours.

### 2.6. Masking

Data were anonymized on the outcome assessor by previously coding the independent variable, which is the access site. However, this was not feasible for participants or investigators who had direct contact with patients and were aware of their catheterization access sites.

### 2.7. Statistical Methods and Additional Analysis

SPSS statistical software version 21 was used to enter and evaluate the data. The intention to treat analysis was used. Continuous variables were summed and provided in the form of a mean and standard deviation, while categorical variables were presented in frequencies and percentages. The Shapiro–Wilk test was used to investigate data for normality. Results with *p* values greater than 0.05 were considered regularly distributed, while those with less than 0.05 were considered not normally distributed. The chi-squared test was used to compare the intervention and control groups for categorical data. Continuous data, on the other hand, were compared using the independent *t*-test when the variable data were normally distributed and the Mann–Whitney *U* test when they were not. When the *p* value was less than 0.05, the significance level was evaluated for the data.

### 2.8. Ethical Consideration

The intervention examined, DRA, has previously been used by many specialists and proven to be safe and successful for cardiac catheterization for diagnostic and interventional procedures [9–11, 16]. The approval was provided by *the Institutional Review Board (IRB) of An-Najah National University* with archive [13] October. This study is registered at ClinicalTrials.gov under the Identifier: NCT04125992.

Patients who met the inclusion criteria and agreed to participate in the study voluntarily were received informed consent and a detailed description of the study's goals, methodology, and potential benefits or risks. Patients who declined to participate in the trial were given the entire treatment plan, which included the standard forearm transradial catheterization and follow-up.

The data obtained in this study were treated with confidentiality. Except for the researchers and those legally permitted, no one had access to the patients' information. If a report on this research or its findings is published, just the conclusions will be shown without exposing the identities of any of the participants.

## 3. Results

### 3.1. Participants Flow and Recruitment

**Figure 3:**
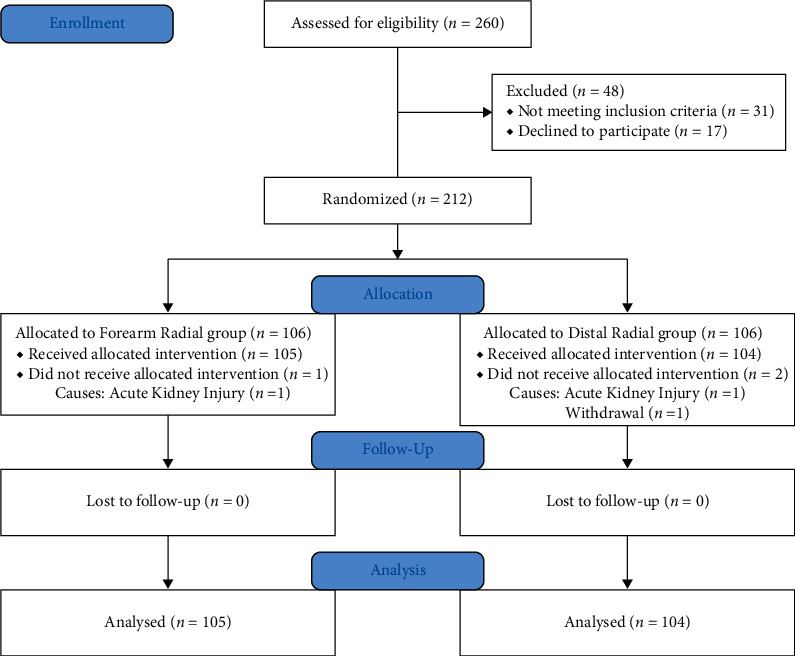
Flow of participants.

The flow of participants is shown in Figure 3.

### 3.2. Baseline Data

Table 1 shows the baseline characteristics of the randomized participants. The groups were comparable in terms of age, gender, weight, and height (age 57.4 ± 10.4, BMI 30.2 ± 5.8, and 155 males (74.1%)). There were no statistically significant differences between the two groups in terms of cardiovascular risk factors, previous cardiac catheterization, and PCI as well as prior vascular approach. In addition, there were no differences between the two groups in terms of cardiac catheterization indication, with unstable angina being the most common in both.

### 3.3. Procedural Outcomes and Side Effects

The procedural data are presented in Tables 2 and 3. Arterial access was successful in 103 (98%) of the FRA patients and 102 (98%) of the DRA patients (*p*=0.992). Two patients in the DRA group experienced access failure (1.9%) related to puncture failure and radial artery spasm. A crossover to the radial artery of the contralateral (right) forearm was needed for these two patients. Radial artery spasm was not observed in the FRA group. However, there were two incidents (1.9%) of access failures in the FRA group. In each of these cases, a crossover to the radial artery of the contralateral (left) forearm was required in each of these cases.

**Figure 4:**
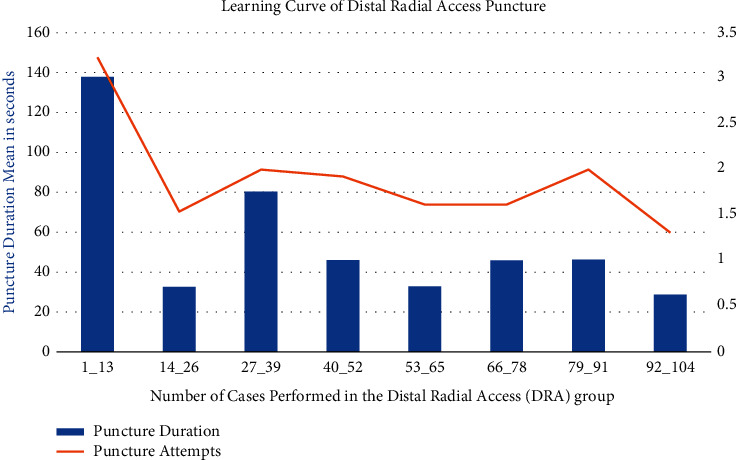
Learning curve of distal radial access puncture.

The number of puncture attempts in this study was considerably higher in the DRA group (1.2 ± 0.60) compared to the FRA group (1.9 ± 1.2, *p* < 0.001). Furthermore, the DRA group had a longer transradial access time (56.3 ± 58.3 s) than the FRA group (20.0 ± 18.4 s, *p* < 0.001). For the puncture time and number of puncture attempts, a learning curve was created.  Figure 4 illustrates a chart of 104 patients divided into eight groups of 13 persons. Each group's mean length and number of puncture attempts were recorded and charted. The learning curve indicated improved puncture duration and a reduction in the number of puncture attempts with time.

Puncture-related discomfort was considerably worse in the DRA group (4 ± 2.2 vs. 3 ± 2.1, *p*=0.001). Coronary interventions (angioplasty or stent) were performed on 39 patients with FRA and 27 DRA patients (*p*=0.082). In DRA and FRA, manual hemostasis was used to achieve hemostasis. The hemostasis time (114 ± 44.4 min) was shorter than that of FRA (134 ± 50.1 min, *p*=0.002). The procedure time, the mean fluoroscopy time, radiation dosage, and contrast volume were considerably lower in the DRA group. Finally, we computed the adjusted *p* value to compensate for the influence of age and PCI (multiple linear regression model adjusted for age and PCI). It was significant for operation duration and fluoroscopy time, indicating that both the variables increased in the FRA group.

Both the groups had an acceptable rate of access site complication. There was no significant bleeding. Minor complications, such as vasospasm, were detected in 3 patients (2.9%) of the FRA group and four patients (3.8%) of the DRA group (*p*=0.729). Access hematoma was more common in the FRA group, although the difference was not statistically significant (*p*=0.313). The most major safety concern, radial artery occlusion at 24 hours, was identified in two patients (1.9%) in the FRA (*p*=0.139). A follow-up Doppler US after 2 weeks revealed a patent radial artery.

## 4. Discussion

### 4.1. Generalizability

Subjects over the age of 18 were evaluated for eligibility, independent of their age, gender, BMI, or past medical issues. The study patients were randomized and had well-balanced characteristics in both the groups. Exclusions from eligibility were implemented to guarantee the safety of STEMI patients and hemodynamically unstable individuals. However, with more operator expertise, these situations may be safely operated by DRA [11, 12]. Furthermore, patients whose access is restricted to the left or right side, radial AVF for hemodialysis and CABG, were omitted to assure randomization. However, DRA can be used for those patients with the accessible hand in normal conditions. In addition, right-sided DRA was effectively used in two patients with the right upper limb pronated 90 degrees from the anatomical position [17–19].

### 4.2. Interpretation, Advantages, and Disadvantages

DRA access is a new technique for cardiac intervention. Many interventional cardiologists highlight the advantages of this access over conventional radial access, notably improved left-sided aortocoronary graft access, potential proximal radial artery sparing, faster hemostasis, low rate of occlusion, and it could be utilized as a possible site for RAO retrograde revascularization. Moreover, the DRA approach is more convenient for the operator and patient. No equipment is needed to maintain the patient's left arm. The operator could work from the patient's right side without leaning over to access the left radial artery, and he can work safely away from the radiation source [7, 16, 20–22]. However, only a few researchers have compared the two approaches, and the published findings are still debatable and restricted.

Given that the DRA is a novel approach and that operators have less experience with it, it is reasonable to expect it to require a longer time and attempts to achieve a successful puncture. A learning curve for the DRA is expected to improve the operator experience over time by reducing the failure rate, puncture number, and puncture duration [10, 11, 14, 23]. This is comparable to the learning curve found when the FRA was first implemented [4, 24,25]. In this study, the learning curve demonstrated improved puncture duration and a reduction in the number of puncture attempts over time (Figure 4).

Our study's puncture success rate was 98%, comparable to prior DRA trials, which demonstrated access rates ranging from 70 to 100% [9–13, 23]. The use of a 6 Fr sheath allowed for easy entry and manipulation for CAG and PCI with simple or complex lesions [10, 11]. Access failure was caused by puncture failure and radial artery spasm. Other reasons for access failure, such as prior radial artery blockage, radial rupture, dissection, and radial tortuosity, were not observed in our study [23].

In our study, the DRA group's procedure duration and fluoroscopy time were substantially reduced, even with the greater rate of PCI in the FRA group. However, the procedure duration and fluoroscopy time were less than the reported average in both the groups [13]. This observation can be explained by the fact that the morphology of the left radial aortic route is similar to the anatomy of the femoral aortic passage, allowing for easier coronary cannulation and catheter manipulations [26].

The increased puncture pain in the DRA at the snuffbox is likely to be caused by needle irritation to the periosteum of the scaphoid and trapezium bones, which are close to the surface [9]. Another anatomical feature associated with puncture pain is that two sensory nerves, the radial nerve and the lateral cutaneous nerve of the forearm, run near the puncture site. Finally, the amount of local anesthetic has a significant impact on DRA puncture pain. As a result, 3–5 ml of 1% xylocaine is recommended [23]. Using the Pearson correlation coefficient, it was found that puncture attempts did not correlate with puncture pain (r (102) = 0.07, *p*=0.476). Likewise, there is no association between puncture duration and puncture pain (r (102) = 0.09, *p*=0.341). This suggests that even though the puncture learning curve reduces the number of attempts and the duration of punctures, pain does not appear to be reduced.

In our study, no serious complications were detected. Minor events such as vasospasm and access hematoma were reported, although the difference was not statistically significant between the two groups. It should be noted that the DRA was associated with numbness in some individuals since the radial nerve is close to the radial artery in the anatomical snuffbox [9, 11, 23]. However, the frequency of this event was not evaluated in this study. Many factors can induce radial artery spasm, including painful stimuli, patient anxiety, and needle stimulation of the artery [23]. Arterial spasm occurred four times in the DRA group, resulting in access failure in one patient. The distal radial artery is smaller in diameter than the proximal radial artery; therefore, it is more susceptible to puncture-mediated vasospasm [9]. However, in the case of DRA vasospasm, we can cross to alternative accesses such as ipsilateral FRA, contralateral DRA, or femoral arteries [9]. Radial spasms can be avoided by administering nitroglycerin or verapamil, minimizing attempts, providing local anesthetic, and avoiding aggressive manipulation [23].

The DRA group had a considerably shorter hemostasis time, which is similar to previously reported findings. This is most probably due to the distal radial artery's smaller diameter, as well as compression of the artery on the bony surface of the scaphoid and trapezium carpal bones, which facilitates and speeds up hemostasis [9, 10, 13, 23].

Radial artery obstruction is the most frequent complication of traditional radial access. The incidence rate of RAO is quite diverse, yet according to Rashid's meta-analysis, the RAO prevalence within 24 hours reached 7.7% [27]. However, the obstruction is reversible in 60% of instances within 1–3 months [28]. Previous studies of DRA showed that local radial artery obstruction within the anatomical snuffbox was reported in only 0–3.1% of patients [6, 7, 29], and forearm radial artery obstruction was found in less than 5% of instances [6].

In our trial, the incidence of RAO was nonsignificant with two proximal radial artery obstruction incidences reported in the FRA group (1.9%) and none in the DRA group (*p*=0.139). The recently released DISCO RADIAL trial similarly showed no significant difference in RAO rates between the two groups [30]. In this study, a strict hemostasis strategy was used to prevent RAO, as well as rigorous operator eligibility requirements, assuring extensive operational experience with both access methods. In our study, we also used the same RAO preventive strategies.

Despite the previously described strategies, the rate of RAO in our study and DISCO RADIAL was even lower in the DRA group, around 2–3 times lower than in the FRA group [30]. This is because the puncture point in the anatomical snuffbox is located beyond the bifurcation into the deep palmar arch. If the distal radial artery is occluded, the flow of the proximal radial artery is maintained via the superficial palmar arch. Therefore, DRA appears to be safe in dialysis patients and cardiac coronary bypass candidates who need a radial graft [31].

## 5. Limitations

In terms of limitations, this study was conducted in a single center (NNUH). The sample size was relatively small to assess all required variables. Patients with STEMI and access side restriction due to prior CABG surgery and AVF were excluded from the study. In our study, we did not employ ultrasound guidance for arterial puncture. However, using ultrasound helps for the identification of anatomical landmarks as well as correct vascular access, which is notably useful in impalpable DRA. Blind puncture, on the other hand, increases the chances of tendon damage and hematoma formation.

## 6. Conclusions

The distal radial approach in cardiac catheterization appears to be a promising technique. It is possible and safe for both coronary angiography and interventions. The success rate is undoubtedly dependent on the patient's selection and the operator's expertise. In our study, a high success rate was achieved, but there was an increase in puncture time, attempts, and pain. However, shorter procedure times, fluoroscopy times, and hemostasis times were reported. A learning curve is expected to improve puncture-related outcomes' overtime. Apparently, this strategy should be included in arterial access choices in the catheterization laboratory, especially in situations when the radial artery must be preserved (hemodialysis, radial graft, etc.). More studies with a larger population after a longer learning period is needed.

## Figures and Tables

**Table 1:** Baseline demographics and clinical characteristics of the participants.

Characteristic	FRA group 105	DRA group 104	*p* value
Age mean (SD)	58.7 (±10.2)	56.1 (±10.7)	0.069^*∗∗*^
BMI mean (SD)	29.6 (±5.5)	30.8 (±6.1)	0.120^*∗∗*^
Gender, male *n* (%)	78 (74.3%)	77 (74.0%)	0.976^*∗*^
Marital status, married *n* (%)	101 (96.2%)	99 (95.2%)	0.722^*∗*^
Smoking, *n* (%)	51 (48.6%)	50 (48.1%)	0.95^*∗*^
Diabetes, *n* (%)	47 (44.8%)	43 (41.3%)	0.618^*∗*^
Hypertension, *n* (%)	56(53.3%)	55 (52.9%)	0.719^*∗*^
Dyslipidemia, *n* (%)	13 (12.4%)	12 (11.5%)	0.851^*∗*^
Cardiovascular diseases, *n* (%)	35 (33.3%)	30 (28.8%)	0.484^*∗*^
Antiplatelets, *n* (%)	72 (68%)	69 (66%)	0.655^*∗*^
Previous Cath, *n* (%)	40 (38.1%)	33 (31.7%)	0.335^*∗*^
Previous Cath site *n* (%)			
None	65 (61.9%)	71 (68.9%)	0.532^*∗*^
Radial	18 (17.1%)	11 (10.7%)	
Femoral	10 (9.5%)	08 (7.8%)	
Femoral and radial	12 (11.4%)	13 (12.6%)	
Previous PCI, *n* (%)	29 (27.6%)	19 (18.3%)	0.108^*∗*^
Indication for Cath. *n* (%)			
Stable angina	14 (13.3%)	23 (21.1%)	0.098^*∗*^
Unstable angina	70 (66.7%)	69 (66.3%)	
NSTEMI	21 (20.0%)	12 (11.5%)	

^*∗*^ Chi-squared test; ^*∗∗*^ independent *t*-test; FRA, forearm radial access; DRA, distal radial access; *n*, frequency; SD, standard deviation; PCI, percutaneous intervention; ACS, acute coronary syndrome; NSTEMI, non-ST-segment elevation myocardial infarction.

**Table 2:** Procedure data and study endpoints.

Endpoint	FRA group 105	DRA group 104	*pvalue*	Adjusted *p* value®
Successful access *n* (%)	103 (98%)	102(98%)	0.0992	
Puncture attempts mean (SD)	1.2 (±0.60)	1.9 (±1.2)	<0.001	<0.001
Puncture attempts > two times	3 (2.9%)	23 (21.1%)	<0.001	
Puncture duration (seconds) mean (SD)	20.0 (±18.4)	56.3 (±58.3)	<0.001	<0.001
Intervention (PCI) *n* (%)	39 (37.1%)	27 (26.0%)	0.082	
Procedure duration (minutes) mean (SD)	18.2 (±15.5)	13.1 (±11.2)	0.006	0.011
Heparin dose, mg mean (SD)	7171.0 (±4818.7)	6100.9 (±2129.2)	0.047	0.172
Fluoroscopy time (minutes) mean (SD)	6.9 (±7.0)	4.4 (±4.2)	0.002	0.003
Radiation dose mean (SD)	1031.8 (±1144.1)	711.2 (±607.6)	0.014	0.063
Contrast dose mean (SD)	72.1 (±47.7)	58.0 (±36.4)	0.015	0.065
Hemostasis time in minutes mean (SD)	134.3 (±50.1)	114.8 (±44.4)	0.002	0.004
Heart rate during procedure mean (SD)	81.4 (±14.7)	78.2 (±9.8)	0.066	
Systolic blood pressure during procedure mean (SD)	128 (±16)	127 (±19)	0.577	
Oxygenation during placement of compression device mean (SD)	96.4 (±1.5)	96.1 (±2.9)	0.457	

^*∗*^ Chi-squared test; ^*∗∗*^ independent *t*-test; ® multiple linear regression model adjusted for age and undergoing PCI; FRA, forearm radial access; DRA, distal radial access; *n*, frequency; SD, standard deviation; PCI, percutaneous intervention.

**Table 3:** Study endpoints.

Endpoint	FRA group 105	DRA group 104	*p* value	Adjusted *p* value®
Access failure (crossover) *n* (%)	2 (1.9%)	2 (1.9%)	0.992	
Access puncture pain mean (SD)	3.0 (±2.1)	4.0 (±2.2)	0.001	0.001
Postprocedural access site pain mean (SD)	1.6 (±1.6)	2.1 (±2.3)	0.081	0.054
Arterial spasm *n* (%)	3 (2.9%)	4 (3.8%)	0.729	
Hematoma *n* (%)	6 (5.8%)	3 (2.8%)	0.313	
Radial artery occlusion in 24 hours *n* (%)	2 (1.9%)	0 (0.0)	0.139	

^*∗*^ Independent *t*-test; ^*∗∗*^ chi-square test; ® multiple linear regression model adjusted for age and undergoing PCI; FRA, forearm radial access; DRA, distal radial access; *n*, frequency; SD, standard deviation.

## Data Availability

All data supporting the study are included in the manuscript or available upon request from the manuscript's corresponding author.

## Abbreviations

DRA: Distal radial access/distal radial artery access
FRA: Forearm radial access/forearm radial artery access
IHD: Ischemic heart disease
ESC: European Society of Cardiology
CAG: Coronary angiography
PCI: Percutaneous coronary intervention
AS: Anatomical snuffbox
NNUH: An-Najah National University Hospital
STEMI: ST-segment elevation myocardial infarction
AVF: Arteriovenous fistula
CABG: Coronary artery bypass grafting
LIMA: Left internal mammary artery
RIMA: Right internal mammary artery
mGy: Milligray
NRS: Numerical rating scale
IRB: Institutional review board
BMI: Body mass index
RAO: Radial artery obstruction.
